# Ten simple rules for biologists learning to program

**DOI:** 10.1371/journal.pcbi.1005871

**Published:** 2018-01-04

**Authors:** Maureen A. Carey, Jason A. Papin

**Affiliations:** 1 Department of Microbiology, Immunology, and Cancer Biology, University of Virginia School of Medicine, Charlottesville, Virginia, United States of America; 2 Department of Biomedical Engineering, University of Virginia, Charlottesville, Virginia, United States of America; Dassault Systemes BIOVIA, UNITED STATES

## Introduction

As big data and multi-omics analyses are becoming mainstream, computational proficiency and literacy are essential skills in a biologist’s tool kit. All “omics” studies require computational biology: the implementation of analyses requires programming skills, while experimental design and interpretation require a solid understanding of the analytical approach. While academic cores, commercial services, and collaborations can aid in the implementation of analyses, the computational literacy required to design and interpret omics studies cannot be replaced or supplemented. However, many biologists are only trained in experimental techniques. We write these 10 simple rules for traditionally trained biologists, particularly graduate students interested in acquiring a computational skill set.

## Rule 1: Begin with the end in mind

When picking your first language, focus on your goal. Do you want to become a programmer? Do you want to design bioinformatic tools? Do you want to implement tools? Do you want to just get these data analyzed already? Pick an approach and language that fits your long- and short-term goals.

**Fig 1:**
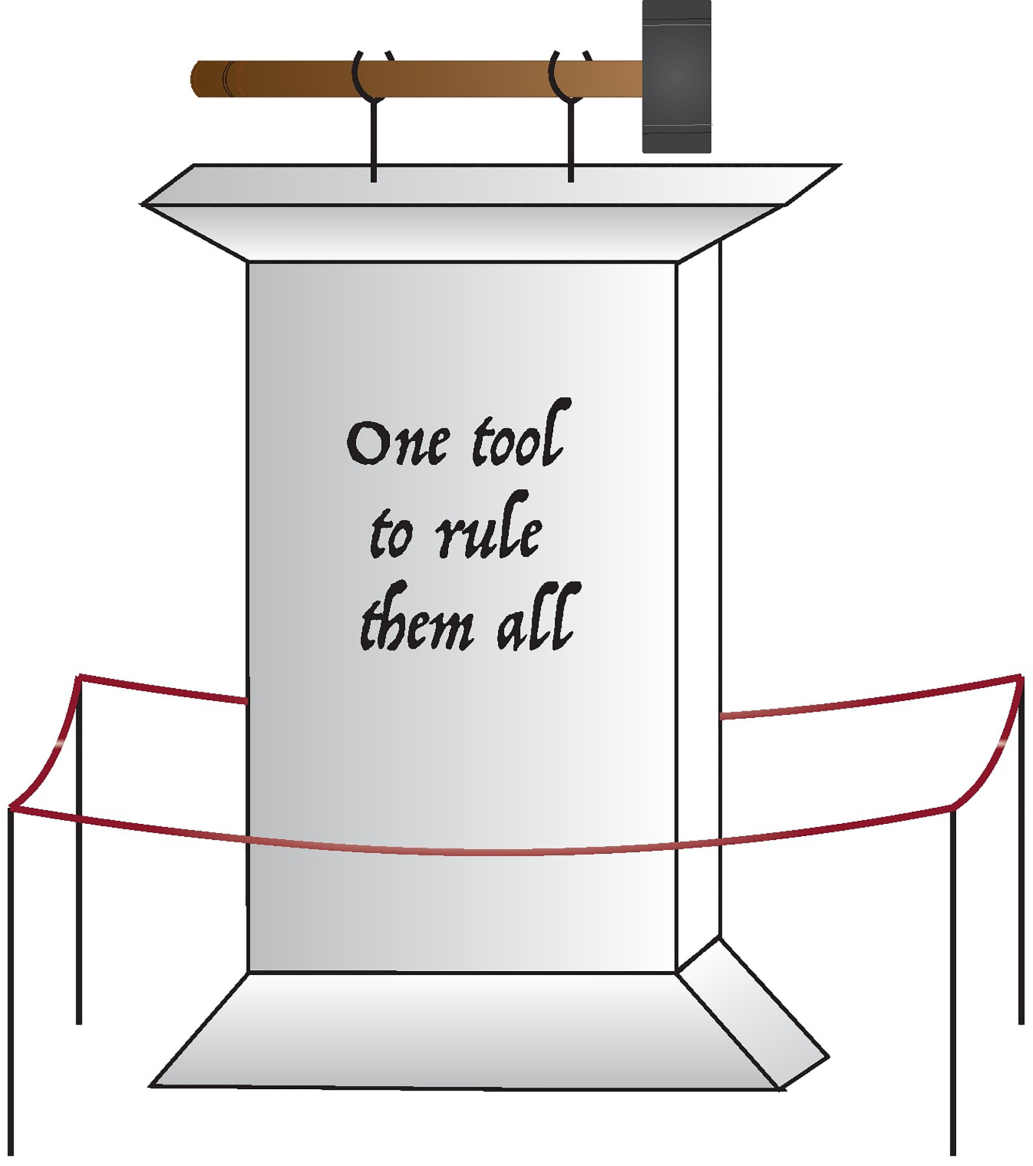
The “one tool to rule them all” (or: how programming languages do not work).

Languages vary in intent and usage. Each language and package was created to solve a particular problem, so there is no universal “best” language (Fig 1). Pick the right tool for the job by choosing a language that is well suited for the biological questions you want to ask. If many people in your field use a language, it likely works well for the types of problems you will encounter. If people in your field use a variety of languages, you have options. To evaluate ease of use, consider how much community support a language has and how many resources that community has created, such as prevalence of user development, package support (documentation and tutorials), and the language’s “presence” on help pages. Practically, languages vary in cost for academic and commercial use. Free languages are more amenable to open source work (i.e., sharing your analyses or packages). See Table 1 for a brief discussion of several programming languages, their key features, and where to learn more.

**Table 1:** A noninclusive discussion of programming languages. A **shell** is a command line (i.e., programming) interface to an operating system, like **Unix** operating systems. **Low-level** programming languages deal with a computer’s hardware. The process of moving from the literal processor instructions toward human-readable applications is called “abstraction.” Low-level languages require little abstraction. **Interpreted** languages are quicker to test (e.g., to run a few lines of code); this facilitates learning through trial and error. Interpreted languages tend to be more human readable. **Compiled** languages are powerful because they are often more efficient and can be used for low-level tasks. However, the distinction between interpreted and compiled languages is not always rigid. All languages presented below are free unless noted otherwise. The Wikipedia page on programming languages provides a great overview and comparison of languages.

Language	Key features	Documentation	Sample tutorials	Community groups
**Bash**	• Most common Unix shell • Practical for execution of scripts written in all other languages • Versatile • Easy to delete files or make other drastic changes • Weaknesses include executing math and limited data structures • Default for macOS and most Linux distributions	• gnu.org/software/bash/manual/ • On macOS’s terminal, type “man <command>” to get the manual for any command (and “q” to exit manual page)	• The Linux Documentation Project’s Beginner’s guide: tldp.org/LDP/Bash-Beginners-Guide/html/ • Ubuntu’s documentation: help.ubuntu.com/community/Beginners/BashScripting • Azet’s GitHub page: github.com/azet/community_bash_style_guide	• Google Plus: plus.google.com/communities/110832059019676429606 • GitHub community resources page: github.com/awesome-lists/awesome-bash
**Python**	• General purpose language • Considered easy to learn due to readability • Flexible syntax considered both a strength and weakness • Interpreted language	• docs.python.org	• Google’s Python class: developers.google.com/edu/python/ • The Hitchhiker’s Guide to Python: docs.python-guide.org/	• Python Users Group: wiki.python.org/moin/LocalUserGroups • Python Special Interest Groups: python.org/community/sigs/
**R**	• Community involvement • Application-focused development • Easy to learn by coupling basic programming and applications • Well-developed visualization • Variable package quality • “Tidy data” community • Interpreted language	• rdocumentation.org • r-project.org • cran.r-project.org	• R for cats: rforcats.net • Books by Hadley Wickham: hadley.nz • R Tutorial’s introduction: r-tutor.com/r-introduction • Cyclismo’s R Tutorial: cyclismo.org/tutorial/R/	• R-Ladies: rladies.org • R Users Group: many
**SAS**	• Statistical computing • High-quality development of statistical functions by commercial and academic developers • Domain-specific usage • Free for students only • Typically a compiled language	• support.sas.com	• Boston University’s SAS Training for Statistics: bu.edu/stat/bu-student-chapter-of-the-asa/sas-training/	• SAS User Groups: sas.com/en_us/connect/user-groups.html
**MATLAB**	• Well-developed applications in engineering • Maintained professionally • Interpreted language • Discounted academic license	• mathworks.com/help/matlab	• Cyclismo’s MATLAB Tutorial: cyclismo.org/tutorial/matlab/ • For purchase courses offered at: matlabacademy.mathworks.com	• MATLAB Central: mathworks.com/matlabcentral/
**Perl**	• General purpose language • Handles text well • Waning community involvement • Syntax modelled after human language • Interpreted language	• perl.org • cpan.org	• Beginning Perl: perl.org/books/beginning-perl/ • Perl maven’s tutorial: perlmaven.com • Perl::Learn: learn.perl.org	• Perl Mongers: pm.org • Perl Monks: perlmonks.org
**Fortran**	• Numeric computation • Fast • Often used for high-performance computing • Limited development • Compiled language	• fortranwiki.org	• many at Fortran wiki: fortranwiki.org/fortran/show/Tutorials	• Fortran Friends: fortran.orpheusweb.co.uk
**C/C++**	• Low-level language • Powerful, used for source code of many other languages • Challenging to learn as it requires explicit syntax • Explicit syntax enforces good programming habits • Compiled language	• devdocs.io/c • cppreference.com	• C programming’s tutorial: cprogramming.com/tutorial/ • Learn-C’s web-based tutorial: learn-c.org	• Standard C++ Foundation: isocpp.org • C/C++ Users Group (CUG): hal9k.com/cug

## Rule 2: Baby steps are steps

Once you’ve begun, focus on one task at a time and apply your critical thinking and problem solving skills. This requires breaking a problem down into steps. Analyzing omics data may sound challenging, but the individual steps do not: e.g., read your data, decide how to interpret missing values, scale as needed, identify comparison conditions, divide to calculate fold change, calculate significance, correct for multiple testing. Break a large problem into modular tasks and implement one task at a time. Iteratively edit for efficiency, flow, and succinctness. Mistakes will happen. That’s ok; what matters is that you find, correct, and learn from them.

## Rule 3: Immersion is the best learning tool

Don’t stitch together an analysis by switching between or among languages and/or point and click environments (Excel [Microsoft; https://www.microsoft.com/en-us/], etc.). While learning, if a job can be done in one language or environment, do it all there. For example, importing a spreadsheet of data (like you would view in Excel) is not necessarily straightforward; Excel automatically determines how to read text, but the method may differ from conventions in other programming languages. If the import process “misreads” your data (e.g., blank cells are not read as blank or “NA,” numbers are in quotes indicating that they are read as text, or column names are not maintained), it can be tempting to return to Excel to fix these with search-and-replace strategies. However, these problems can be fixed by correctly reading the data and by understanding the language’s data structures. Just like a spoken language [1, 2], immersion is the best learning tool [3, 4]. In addition to slowing the learning curve, transferring across programs induces error. See References [5–7] for additional Excel or word processing–induced errors.

Eventually, you may identify tasks that are not well suited to the language you use. At that point, it may be helpful to pick up another language in order to use the right tool for the job (see Rule 1). In fact, understanding one language will make it easier to learn a second. Until then, however, focus on immersion to learn.

## Rule 4: Phone a friend

There are numerous online resources: tutorials, documentation, and sites intended for community Q and A (StackOverflow, StackExchange, Biostars, etc.), but nothing replaces a friend or colleague’s help. Find a community of programmers, ranging from beginning to experienced users, to ask for help. You may want to look for both technical support (i.e., a group centered around a language) and support regarding a particular scientific application (e.g., a group centered around omics analyses). Many universities have scientific computing groups, housed in the library or information technology (IT) department; these groups can be your starting point. If your lab or university does not have a community of programmers, seek them out virtually or locally. Coursera courses, for example, have comment boards for students to answer each other’s questions and learn from their peers. Organizations like Software and Data Carpentry or language user groups have mailing lists to connect members. Many cities have events organized by language-specific user groups or interest groups focused on big data, machine learning, or data visualization. These can be found through meetup.com, Google groups, or through a user group’s website; some are included in Table 1.

Once you find a community, ask for help. At the beginning stages, in-person help to deconstruct or interpret an online answer is invaluable. Additionally, ask a friend for code. You wouldn’t write a paper without first reading a lot of papers or begin a new project without shadowing a few experimenters. First, read their code. Implement and interpret, trying to understand each line. Return to discuss your questions. Once you begin writing, ask for edits.

## Rule 5: Learn how to ask questions

**Fig 2:**
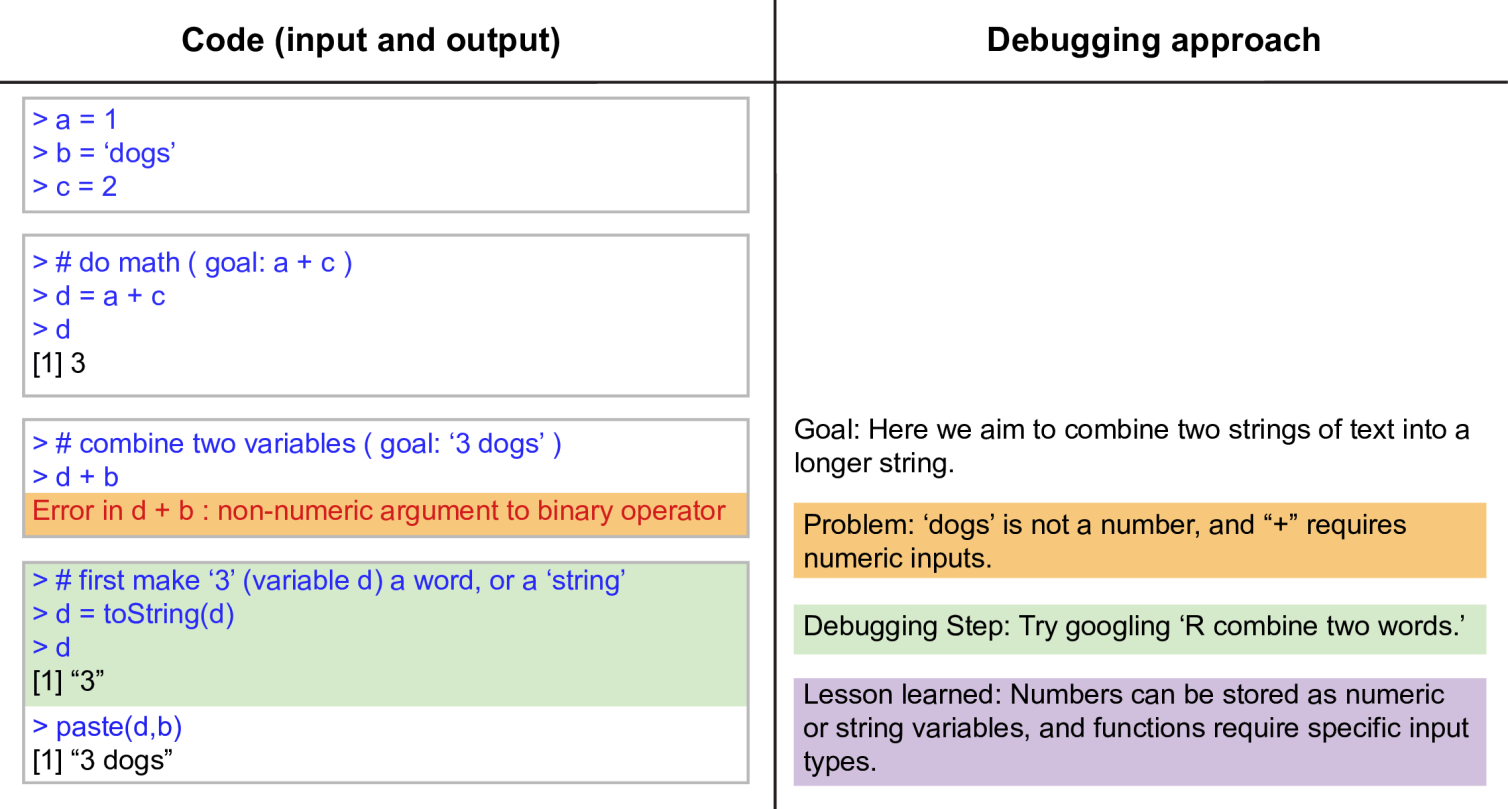
Anatomy of an error message, Part 1 (or: How to write more than one line of code). Here we show an example of the debugging process in R using the RStudio environment, with the goal of concatenating two words.

**Fig 3:**
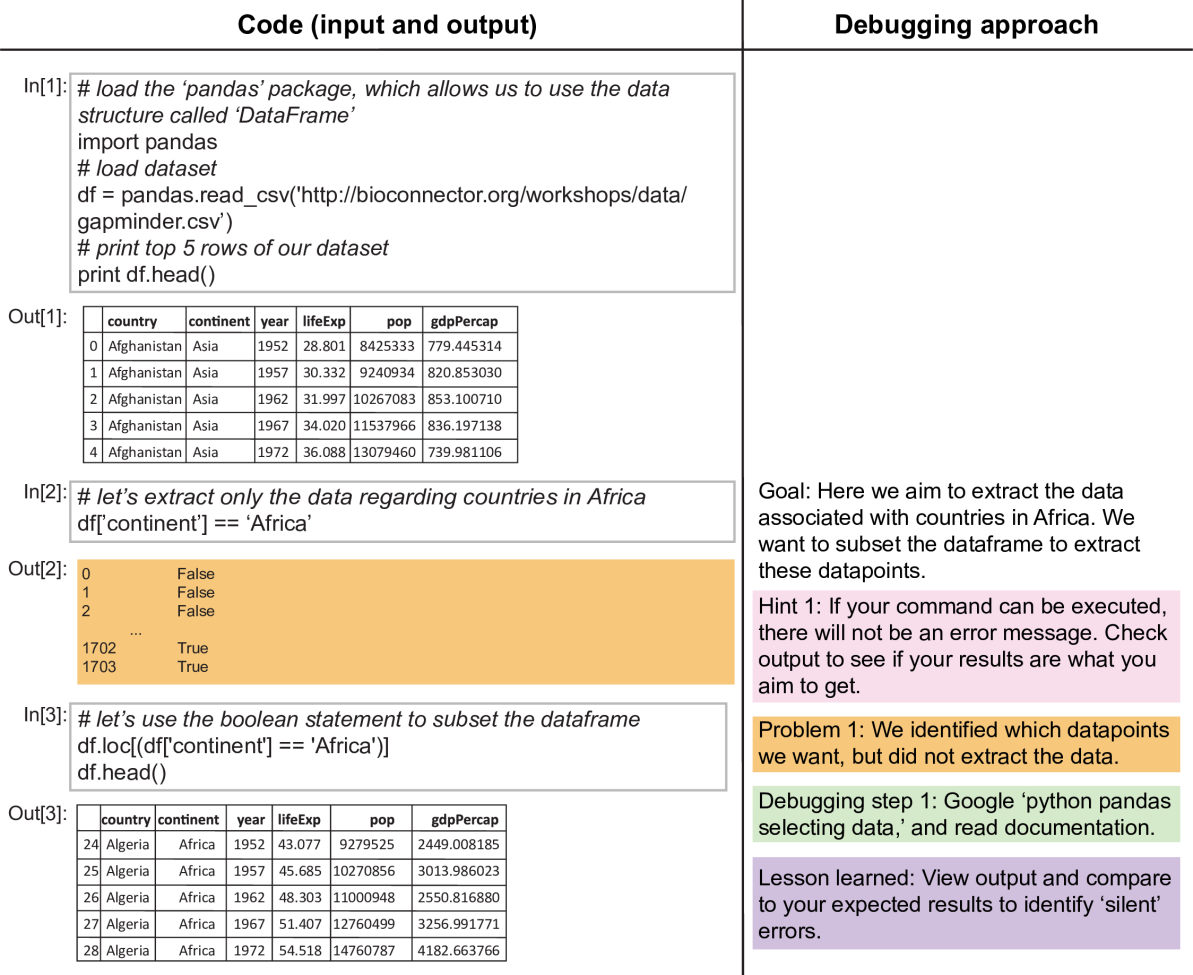
Anatomy of an error message, Part 2 (or: Just because it works, doesn’t mean it’s right). Here we provide more examples of the debugging process. Examples shown in Figs 3–5 are conducted in Python using a Jupyter notebook. Environments like RStudio (in Fig 2) and Jupyter notebooks are two examples of integrated development environments; these environments offer additional support, including built-in debugging tools. First, we show an error that does not induce an error message, but the user must debug nonetheless.

**Fig 4:**
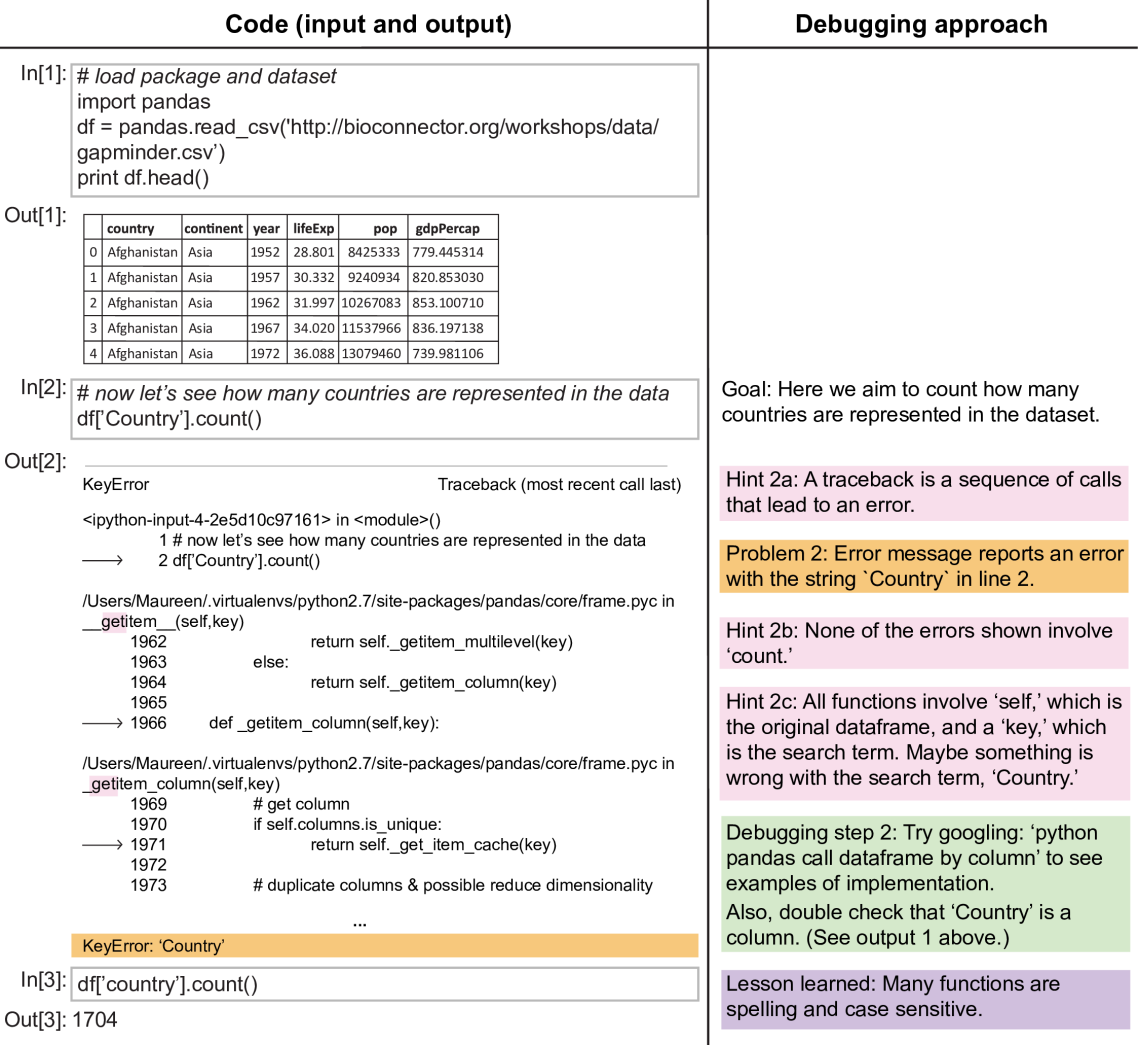
Anatomy of an error message, Part 3 (or: Trace your way back to the problem). Here we show an explicit error message.

**Fig 5:**
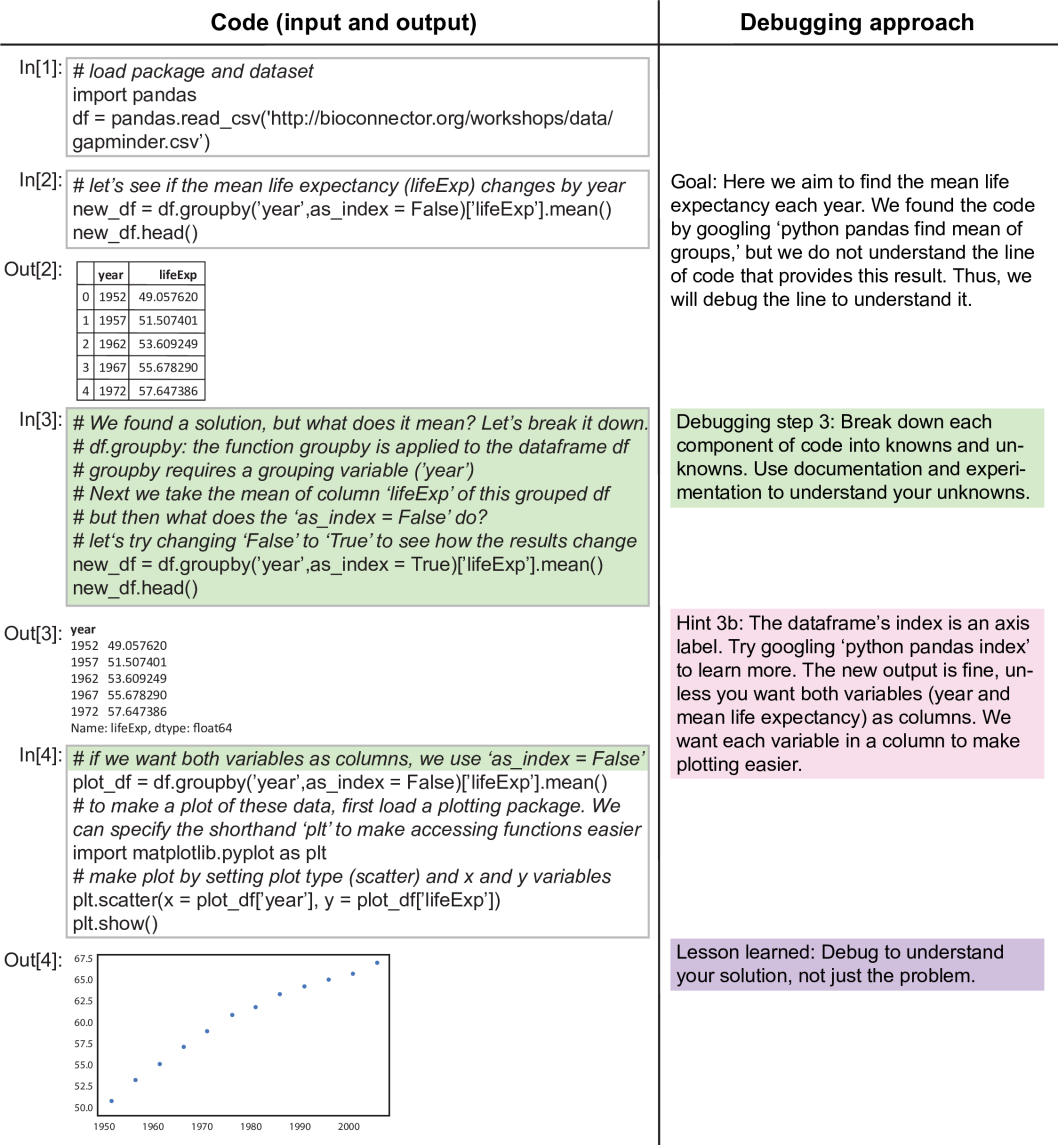
Anatomy of an error message, Part 4 (or: Debugging a solution). Lastly, we show how to debug a solution to understand a line of code found on the internet.

There’s an answer to almost anything online, but you have to know what to ask to get help. In order to know what to ask, you have to understand the problem. Start by interpreting an error message. Watch for generic errors and learn from them. Identify which component of your error message indicates what the issue is and which component indicates where the issue is (Figs 2–5). Understanding the problem is essential; this process is called “debugging.” Without truly understanding the problem, any “solution” will ultimately propagate and escalate the mistake, making harder-to-interpret errors down the road. Once you understand the problem, look for answers. Looking for answers requires effective googling. Learn the vocabulary (and meta-vocabulary) of the language and its users. Once you understand the problem and have identified that there is no obvious (and publicly available) solution, ask for answers in programming communities (see Rule 4 and Table 1). When asking, paraphrase the fundamental problem. Include error messages and enough information to reproduce the problem (include packages, versions, data or sample data, code, etc.). Present a brief summary of what was done, what was intended, how you interpret the problem, what troubleshooting steps were already taken, and whether you have searched other posts for the answer.

See the following website for suggestions: http://codereview.stackexchange.com/help/how-to-ask and [8]. End with a “thank you” and wait for the help to arrive.

## Rule 6: Don’t reinvent the wheel

Rule 6 can also be found in “Ten Simple Rules for the Open Development of Scientific Software” [9], “Ten Simple Rules for Developing Public Biological Databases” [10], “Ten Simple Rules for Cultivating Open Science and Collaborative R&D” [11], and “Ten Simple Rules To Combine Teaching and Research” [12]. Use all resources available to you, including online tutorials, examples in the language’s documentation, published code, cool snippets of code your labmate shared, and, yes, your own work. Read widely to identify these resources. Copy-and-paste is your friend. Provide credit if appropriate (i.e., comment “adapted from so-n-so’s X script”) or necessary (e.g., read through details on software licenses). Document your scripts by commenting in notes to yourself so that you can use old code as a template for future work. These comments will help you remember what each line of code intends to do, accelerating your ability to find mistakes.

## Rule 7: Develop good habits early on

Computational research is research, so use your best practices. This includes maintaining a computational lab notebook and documenting your code. A computational lab notebook is by definition a lab notebook: your lab notebook includes protocols, so your computational lab notebook should include protocols, too. Computational protocols are scripts, and these should include the code itself and how to access everything needed to implement the code. Include input (raw data) and output (results), too. Figures and interpretation can be included if that’s how you organize your lab notebook. Develop computational “place habits” (file-saving strategies). It is easier to organize one drawer than it is to organize a whole lab, so start as soon as you begin to learn to program. If you can find that experiment you did on June 12, 2011—its protocol and results—in under five minutes, you should be able to find that figure you generated for lab meeting three weeks ago, complete with code and data, in under five minutes as well. This requires good version control or documentation of your work. Like with protocols, each time you run a script, you should note any modifications that are made. Document all changes in experimental and computational protocols. These habits will make you more efficient by enhancing your work’s reproducibility. For specific advice, see “Ten Simple Rules for a Computational Biologist’s Laboratory Notebook” [13], “Ten Simple Rules for Reproducible Computational Research” [14], and “Ten Simple Rules for Taking Advantage of Git and GitHub” [15].

## Rule 8: Practice makes perfect

Use toy datasets to practice a problem or analysis. Biological data get big, fast. It’s hard to find the computational needle-in-a-haystack, so set yourself up to succeed by practicing in controlled environments with simpler examples. Generate small toy datasets that use the same structure as your data. Make the toy data simple enough to predict how the numbers, text, etc., should react in your analysis. Test to ensure they do react as expected. This will help you understand what is being done in each step and troubleshoot errors, preparing you to scale up to large, unpredictable datasets. Use these datasets to test your approach, your implementation, and your interpretation. Toy datasets are your negative control, allowing you to differentiate between negative results and simulation failure.

## Rule 9: Teach yourself

**Fig 6:**
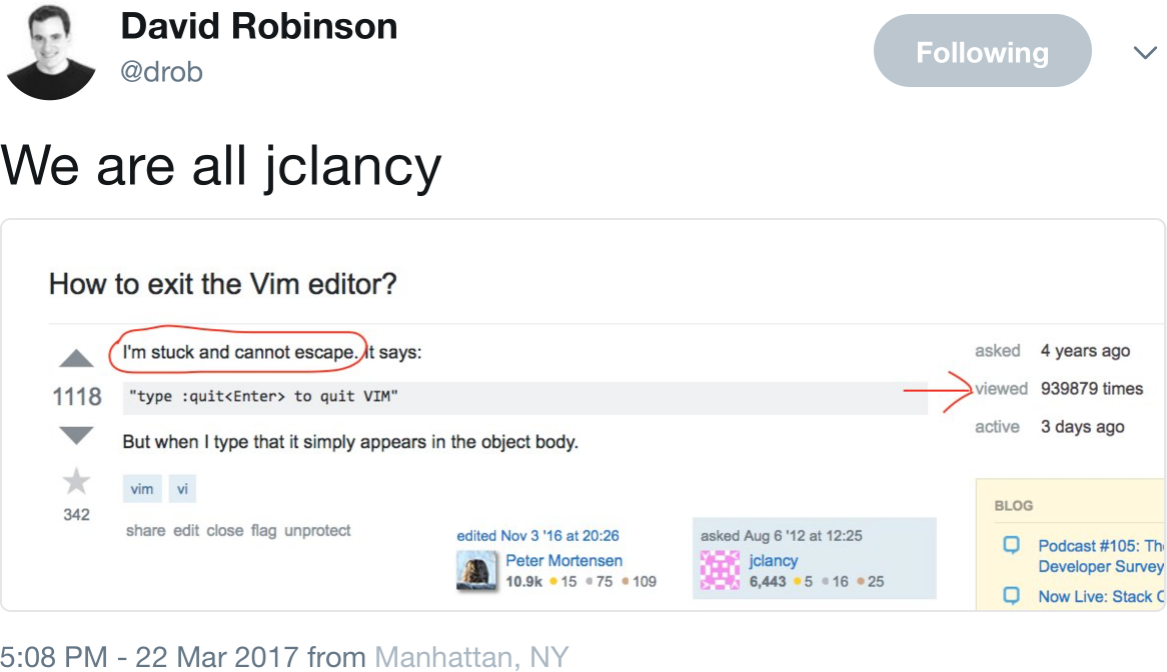
“How to exit the vim editor?” (or: We all get stuck at some point). Now viewed >1.33 million times; see: http://stackoverflow.com/questions/11828270/how-to-exit-the-vim-editor.

How would you teach you if you were another person? You would teach with a little more patience and a bit more empathy than you are practicing now. You are not alone in your occasional frustration (Fig 6). Learning takes time, so plan accordingly. Introductory courses are helpful to learn the basics because the basics are easy to neglect in self-study. Articulate clear expectations for yourself and benchmarks for success. Apply some of the structure (deadlines, assignments, etc.) you would provide a student to help motivate and evaluate your progress. If something isn’t working, adjust; not everyone learns best by any one approach. Explore tutorials, online classes, workshops, books like *Practical Computing for Biologists* [16], local programming meetups, etc., to find your preferred approach.

## Rule 10: Just do it

Just start coding. You can’t edit a blank page.

Learning to program can be intimidating. The power and freedom provided in conducting your own computational analyses bring many decisions points, and each decision brings more room for mistakes. Furthermore, evaluating your work is less black-and-white than for some experiments. However, coding has the benefit that failure is risk free. No resources are wasted—not money, time (a student’s job is to learn!), or a scientific reputation. In silico, the playing field is leveled by hard work and conscientiousness. So, while programming can be intimidating, the most intimidating step is starting.

## Conclusion

Markowetz recently wrote, “Computational biologists are just biologists using a different tool” [17]. If you are a traditionally trained biologist, we intend these 10 simple rules as instruction (and pep talk) to learn a new, powerful, and exciting tool. The learning curve can be steep; however, the effort will pay dividends. Computational experience will make you more marketable as a scientist (see “Top N Reasons To Do A Ph.D. or Post-Doc in Bioinformatics/Computational Biology” [18]). Computational research has fewer overhead costs and reduces the barrier to entry in transitioning fields [19], opening career doors to interested researchers. Perhaps most importantly, programming skills will make you better able to implement and interpret your own analyses and understand and respect analytical biases, making you a better experimentalist as well. Therefore, the time you spend at your computer is valuable. Acquiring programming expertise will make you a better biologist.
